# Contemporary clinical and economic outcomes among oral anticoagulant treated and untreated elderly patients with atrial fibrillation: Insights from the United States Medicare database

**DOI:** 10.1371/journal.pone.0263903

**Published:** 2022-02-17

**Authors:** Muhammad Bilal Munir, Patrick Hlavacek, Allison Keshishian, Jennifer D. Guo, Rajesh Mallampati, Mauricio Ferri, Cristina Russ, Birol Emir, Matthew Cato, Huseyin Yuce, Jonathan C. Hsu

**Affiliations:** 1 Section of Electrophysiology, Division of Cardiology, University of California San Diego School of Medicine, La Jolla, CA, United States of America; 2 Pfizer, New York, NY, United States of America; 3 STATinMED Research, LLC, Ann Arbor, MI, United States of America; 4 Bristol-Myers Squibb, Lawrenceville, NJ, United States of America; 5 New York City College of Technology, City University of New York, New York, NY, United States of America; Hualien Tzu Chi Hospital Buddhist Tzu Chi Medical Foundation, TAIWAN

## Abstract

**Background:**

Oral anticoagulants (OACs) mitigate the risk of stroke in atrial fibrillation (AF) patients.

**Objective:**

Elderly AF patients who were treated with OACs (apixaban, dabigatran, edoxaban, rivaroxaban, or warfarin) were compared against AF patients who were not treated with OACs with respect to their clinical and economic outcomes.

**Methods:**

Newly diagnosed AF patients were identified between January 2013 and December 2017 in the Medicare database. Evidence of an OAC treatment claim on or after the first AF diagnosis was used to classify patients into treatment-defined cohorts, and these cohorts were further stratified based on the initial OAC prescribed. The risks of stroke/systemic embolism (SE), major bleeding (MB), and death were analyzed using inverse probability treatment weighted time-dependent Cox regression models, and costs were compared with marginal structural models.

**Results:**

The two treatment groups were composed of 1,421,187 AF patients: OAC treated (N = 583,350, 41.0% [36.4% apixaban, 4.9% dabigatran, 0.1% edoxaban, 26.7% rivaroxaban, and 31.9% warfarin patients]) and untreated (N = 837,837, 59.0%). OAC-treated patients had a lower adjusted risk of stroke/SE compared to untreated patients (hazard ratio [HR]: 0.70; 95% confidence interval [CI]: 0.68–0.72). Additionally patients receiving OACs had a lower adjusted risk of death (HR: 0.56; 95% CI: 0.55–0.56) and a higher risk of MB (HR: 1.57; 95% CI: 1.54–1.59) and this trend was consistent across each OAC sub-group. The OAC-treated cohort had lower adjusted total healthcare costs per patient per month ($4,381 vs $7,172; p < .0001).

**Conclusion:**

For the OAC-treated cohort in this elderly US population, stroke/SE and all-cause death were lower, while risk of MB was higher. Among OAC treated patients, total healthcare costs were lower than those of the untreated cohort.

## Introduction

Atrial fibrillation (AF) is the most common cardiac arrhythmia encountered in clinical practice with an estimated US prevalence of 700–775 per 100,000 in the year 2010 [1]. AF is associated with an increased risk of stroke, and strokes related to AF have worse outcomes when compared to strokes not related to AF [2, 3]. AF also confers an increased risk of death as demonstrated by a sub-analysis of the Framingham Heart Study which found nearly a 1.5–1.9-fold mortality risk in patients who developed AF during the 40 years of follow-up after adjusting for other pre-existing cardiovascular co-morbidities [4]. Additionally, AF is associated with significant increased national healthcare costs ranging from $6-$26 billion shown by a recent US study from the MarketScan Commercial and Medicare database when compared to a propensity score matched non-AF control group [5].

Earlier studies have shown significant underutilization of oral anti-coagulants (OACs) in AF patients at high risk of stroke [6–9]. This low utilization has persisted even after the introduction of direct acting oral anti-coagulants (DOACs) despite their ease of use by the patients. Although earlier studies have assessed clinical outcomes such as stroke and death in AF patients based on specific OAC treatment [10–12], most have not evaluated a large contemporary cohort of AF patients treated mostly with a DOAC therapy. Additionally, earlier studies on healthcare associated costs did not stratify AF patients based on specific OAC treatment [13–15] and were not performed when DOAC therapy was the predominant treatment strategy. The purpose of our study was to assess clinical and economic outcomes in a contemporary real-world cohort of AF patients who were treated with an OAC (warfarin, apixaban, dabigatran, edoxaban, and rivaroxaban) versus those who were not after their first AF diagnosis.

## Methods

### Data source

This was a retrospective cohort study conducted using the United States Centers for Medicare & Medicaid Services (CMS) fee-for-service Medicare database from January 2012 until December 2017. Fee-for-service Medicare is a federal health insurance program which covers over 38 million patients, including those aged ≥65 years and other special groups of patients in the United States [16]. The database contains medical and pharmacy claims from Medicare data, including inpatient, outpatient, carrier, Part D, skilled nursing facility, home health agency, and durable medical equipment claims. Pharmacy claims are recorded based on the drug dispensed using the National Drug Code coding system.

### Patient selection

**Fig 1:**
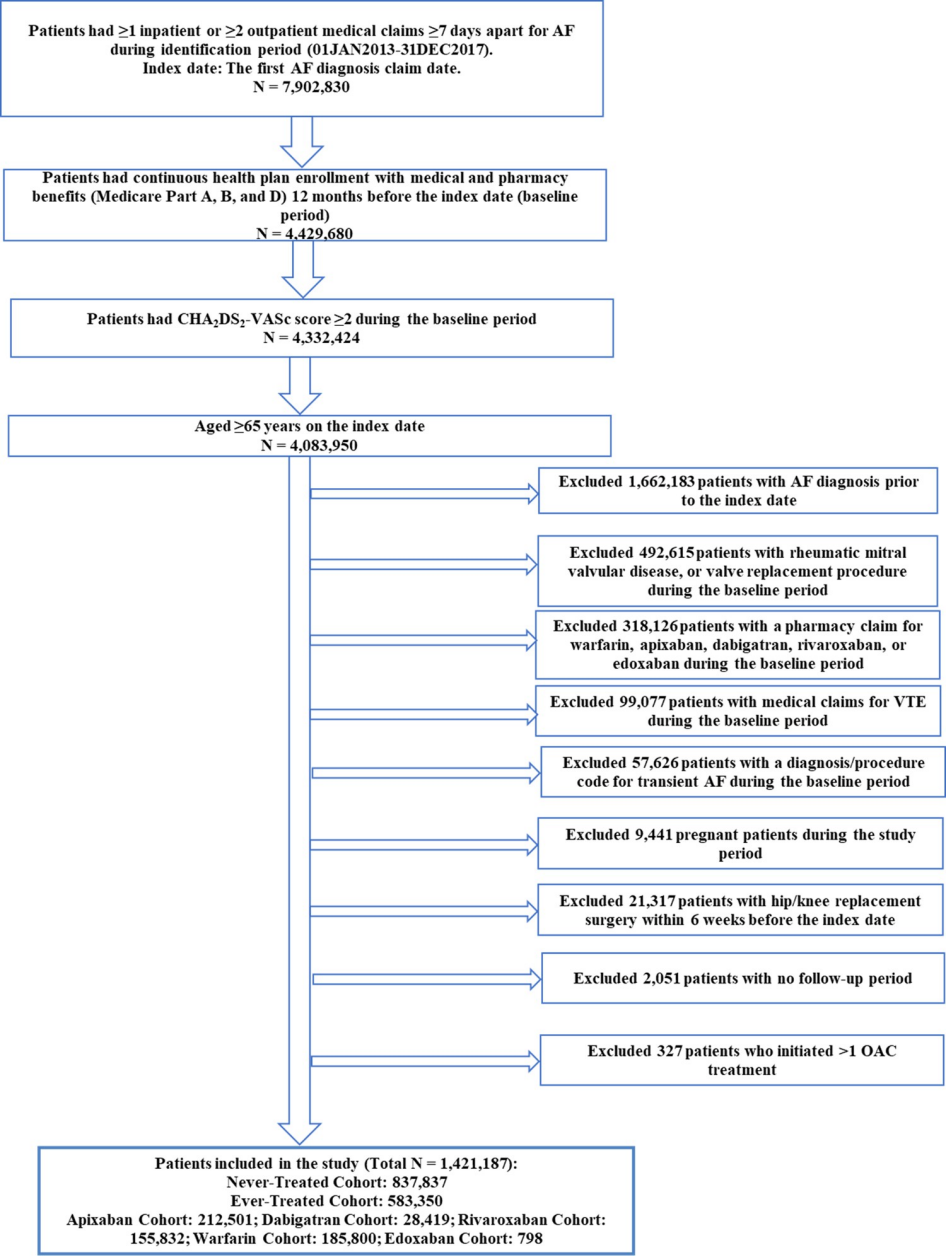
Patient selection criteria. AF- Atrial Fibrillation; CHA2DS2-VASc- Congestive heart failure, Hypertension, Age ≥ 75 years, Diabetes mellitus, Stroke, Vascular disease, Age 65–74 years, Sex category; ICD-9/10-CM.

Incident NVAF patients with a high risk of stroke treated and not treated with OACs were studied. Patients with ≥1 inpatient or ≥2 outpatient medical claims for AF (≥ 7 days apart and in any diagnosis position) between January 2013 and December 2017 were selected (index date: first AF diagnosis claim date). Patients were required to have continuous enrollment for Medicare Parts A, B, and D plans and a CHA_2_DS_2_-VASc score ≥ 2 during the baseline period of 12 months before the index date. Patients were required to be 65 years or older on the index date. Patients were excluded if they had: 1) pharmacy claims for OACs (warfarin, apixaban, dabigatran, rivaroxaban, or edoxaban), an AF diagnosis, or medical claims indicating a diagnosis of rheumatic mitral valvular heart disease, valve replacement procedure, venous thromboembolism, or transient AF during the baseline period; 2) pregnancy claims during the study period; 3) hip/knee replacement surgery within the 6 weeks prior to or on the index date; 4) no follow-up; or 5) >1 OAC treatment on the index date (Fig 1).

### Cohort definition

Follow-up period was the duration from the index date until the earliest of death, disenrollment, end of study, or index OAC discontinuation (if treated with OAC). This follow-up period was required to be >0 days. OAC treatment was evaluated on or after the index date during the follow-up period. Patients were considered OAC treated on the first date of OAC prescription and were assigned to the individual OAC cohorts (apixaban, dabigatran, rivaroxaban, and warfarin). Patients without OAC treatment during the follow-up period were assigned to the OAC untreated cohort.

### Baseline variables

Demographic variables including age, sex, race, and geographic region were evaluated on the index date. Charlson comorbidity index, CHA_2_DS_2_-VASc score [17], modified HAS-BLED score [18], bleeding, stroke/systemic embolism (SE), and comorbidities such as obesity, congestive heart failure, diabetes, hypertension, chronic obstructive pulmonary disease, renal disease, myocardial infarction, dyspepsia, peripheral vascular disease, transient ischemic disease, coronary artery disease, and history of falls were examined during the baseline period.

### Study outcomes

Clinical outcomes including stroke/SE, major bleeding, and death were evaluated during the follow-up period. Stroke/SE and major bleeding were based on hospitalizations with stroke/SE or major bleeding as the principal diagnosis [19, 20]. Selected sub-categories of stroke/SE (ischemic stroke) and major bleeding (intracranial hemorrhage [ICH], and gastrointestinal [GI] bleeding) were evaluated using a similar approach. The data on all-cause mortality was obtained by validated Social Security records that included the date of death [21].

Healthcare costs were evaluated during the follow-up period and included all-cause healthcare costs in the following settings: inpatient, outpatient, pharmacy, and other settings (durable medical equipment, home health agency, hospice, skilled nursing facility). In addition, stroke/SE and major bleeding-related hospitalization costs were calculated from the admission to discharge date of the first stroke/SE or major bleeding hospitalization. Healthcare costs indicated above were calculated per patient per month (PPPM) and adjusted to 2017 US dollars.

### Statistical analysis

For the descriptive analysis, two fixed cohorts of OAC treated vs OAC untreated were created. Student’s t-test and Chi-square tests were used to compare the study variables between the study cohorts. The OAC treated cohort was further stratified by the index OAC type. A p-value<0.05 was considered statistically significant.

Stabilized inverse probability treatment weighting (IPTW) was conducted to balance patient characteristics between the OAC treatment cohorts (OAC treated vs OAC untreated, apixaban vs OAC untreated, dabigatran vs OAC untreated, rivaroxaban vs OAC untreated, and warfarin versus OAC untreated). Propensity scores, which estimate the average treatment effect, were derived using a logistic model, in which propensity score model covariates were demographics, comorbidities, medications, and baseline hospitalization [22]. After the propensity score calculation for each cohort, each patient was weighted by the inverse of the probability of their treatment option (treated: weight = 1/propensity score; untreated: weight = 1/(1-propensity score)). In the weighted population, the baseline characteristics were well balanced across all cohort comparisons.

After IPTW, the multivariable time-varying cox proportional hazard models were used to assess the risk of stroke/SE, major bleeding, and mortality comparing the treatment group of interest with the OAC untreated group being the reference. In these hazard models, there were three time-varying variables, OAC treatment (a patient could change from the untreated to any one of the treated cohort subtypes), and CHA_2_DS_2_-VASc and modified HAS-BLED scores.

To compare healthcare costs between treatment cohorts and the untreated OAC cohort, marginal structural models were used with treatment status as a time-varying variable [23]. Patient demographics and clinical characteristics were adjusted in the weights, which were allowed to change with treatment status. Weights were computed using the treatment and censoring weights for the treated and untreated periods of the ever-treated OAC cohort. Healthcare costs during the corresponding periods were evaluated. To maintain consistency with the treated cohort, similar weights were computed for the two equal time periods created by splitting the follow-up period of the OAC untreated cohort. The marginal structure model included a generalized linear model with gamma distribution and log link.

Three sensitivity analyses were also conducted. The first sensitivity analysis included all-cause death as a competing event to the clinical outcomes (stroke/SE and major bleeding). The second sensitivity analysis censored the follow-up at the earliest of follow-up end or one year. The third sensitivity analysis assessed the presence of residual confounding by evaluating falsification outcomes that were not associated with OAC treatment using the same methodology as the main analysis. The falsification outcomes included chronic obstructive pulmonary disease (COPD), pneumonia and urinary tract infection (UTI) hospitalizations [24, 25].

Data analysis was performed using statistical software SAS version 9.4 (SAS Institute Inc., Cary, NC, USA). Since this study did not involve the collection, use, or transmittal of individually identifiable data, it was exempt from Institutional Review Board review. Both the datasets and the security of the offices where analysis was completed (and where the datasets are kept) meet the requirements of the Health Insurance Portability and Accountability Act of 1996.

## Results

After applying the selection criteria, 1,421,187 AF patients were selected. Of the included patients, 583,350 patients (41.0%) were treated with an OAC and the average time to OAC treatment was 144 days from the index AF date. Of the OAC treated patients, 212,501 (36.4%) were treated with apixaban, 28,419 (4.9%) with dabigatran, 798 (0.1%) with edoxaban, 155,832 (26.7%) with rivaroxaban, and 185,800 (31.9%) were treated with warfarin (Fig 1).

Prior to weighting, the average age was 81.3 years for the OAC untreated cohort and 78.0 years for the OAC treated cohort (78.4 years for apixaban, 77.0 years for dabigatran, 77.3 years for rivaroxaban, and 78.3 years for warfarin cohort). The CHA_2_DS_2_-VASc (4.5 vs 4.8; p<0.0001) and HAS-BLED scores (3.2 vs 3.5; p<0.0001) during the baseline period were lower in the OAC treated compared with the OAC untreated cohort. Baseline bleeding (19.3% vs 29.2%; p<0.0001), stroke/SE (11.3% vs 14.3%; p<0.0001), and congestive heart failure (26.5% vs 34.7%; p<0.0001) were less prevalent in the OAC treated cohort compared with the OAC untreated cohort. Obesity was more frequent in the OAC treated cohort compared with the OAC untreated cohort (21.2% vs 15.3%; p<0.0001). In general, consistent trends were observed in the individual OAC cohorts compared with the OAC untreated cohort (Table 1).

**Table 1:** Baseline descriptive results for ever-treated and OAC untreated with OAC cohorts.

	Ever-Treated with OAC Cohort*N = 583,350		Ever-Treated With OAC Cohort^†^								OAC Untreated Cohort* N = 837,837
			Apixaban Cohort N = 212,501		Dabigatran Cohort N = 28,419		Rivaroxaban Cohort N = 155,832		Warfarin Cohort N = 185,800		

	%/Mean (SD)	p-value	%/Mean (SD)	p-value	%/Mean (SD)	p-value	%/Mean (SD)	p-value	%/Mean (SD)	p-value	%/Mean (SD)
**Age**	78.0 (7.3)	< .0001	78.4 (7.4)	< .0001	77.0 (7.0)	< .0001	77.3 (7.1)	< .0001	78.3 (7.4)	< .0001	81.3 (8.8)
65–74 years	36.0%	< .0001	34.3%	< .0001	40.8%	< .0001	39.3%	< .0001	34.4%	< .0001	26.7%
75–84 years	43.0%	< .0001	42.8%	< .0001	42.6%	< .0001	42.8%	< .0001	43.3%	< .0001	34.5%
≥ 85 years	21.1%	< .0001	22.9%	< .0001	16.5%	< .0001	17.9%	< .0001	22.3%	< .0001	38.8%
**Sex**											
Male	45.6%	< .0001	44.4%	< .0001	48.0%	< .0001	46.8%	< .0001	45.7%	< .0001	42.6%
Female	54.4%	< .0001	55.6%	< .0001	52.0%	< .0001	53.2%	< .0001	54.3%	< .0001	57.4%
**Race**											
White	90.0%	< .0001	90.6%	< .0001	90.1%	< .0001	90.2%	< .0001	89.3%	< .0001	86.4%
Black	5.1%	< .0001	4.7%	< .0001	4.2%	< .0001	4.5%	< .0001	6.1%	< .0001	7.6%
Others	4.9%	< .0001	4.7%	< .0001	5.7%	0.0150	5.3%	< .0001	4.6%	< .0001	6.0%
**US Geographic Region**											
Northeast	19.7%	< .0001	19.4%	< .0001	21.9%	< .0001	19.2%	< .0001	20.1%	< .0001	18.5%
North Central	26.9%	< .0001	23.2%	< .0001	24.1%	0.5649	24.9%	< .0001	33.2%	< .0001	24.3%
South	37.0%	< .0001	42.1%	< .0001	37.3%	< .0001	38.5%	< .0001	29.8%	< .0001	39.1%
West	16.3%	< .0001	15.1%	< .0001	16.4%	< .0001	17.2%	< .0001	16.7%	< .0001	17.8%
Other	0.2%	< .0001	0.1%	< .0001	0.2%	0.6914	0.2%	0.1144	0.2%	0.7684	0.2%
**Medicaid Dual Eligibility**	23.4%	< .0001	20.9%	< .0001	22.8%	< .0001	22.7%	< .0001	27.2%	< .0001	34.5%
**Part-D low income subsidy**	26.2%	< .0001	23.5%	< .0001	25.7%	< .0001	25.2%	< .0001	30.1%	< .0001	37.4%
**Deyo-Charlson Comorbidity Index Score**	2.9 (2.6)	< .0001	2.9 (2.6)	< .0001	2.5 (2.4)	< .0001	2.6 (2.4)	< .0001	3.2 (2.7)	< .0001	3.8 (3.1)
**CHA**_**2**_ **DS**_**2**_ **-VASc Score**	4.5 (1.6)	< .0001	4.5 (1.6)	< .0001	4.3 (1.5)	< .0001	4.3 (1.5)	< .0001	4.7 (1.6)	< .0001	4.8 (1.6)
2–3	28.8%	< .0001	28.5%	< .0001	33.8%	< .0001	33.1%	< .0001	24.9%	< .0001	21.7%
4–5	46.0%	< .0001	46.3%	< .0001	45.2%	0.7927	45.5%	0.2461	46.2%	< .0001	45.3%
≥6	25.2%	< .0001	25.2%	< .0001	21.0%	< .0001	21.4%	< .0001	28.9%	< .0001	33.0%
**HAS-BLED Score** ^**‡**^	3.2 (1.2)	< .0001	3.2 (1.2)	< .0001	3.1 (1.1)	< .0001	3.1 (1.2)	< .0001	3.3 (1.2)	< .0001	3.5 (1.3)
0–2	30.4%	< .0001	29.6%	< .0001	34.7%	< .0001	33.3%	< .0001	28.2%	< .0001	22.9%
3–4	54.5%	0.0021	55.1%	< .0001	53.8%	0.0974	54.4%	0.2423	54.1%	0.2471	54.3%
≥5	15.1%	< .0001	15.3%	< .0001	11.6%	< .0001	12.3%	< .0001	17.7%	< .0001	22.9%
**Prior bleed**	19.3%	< .0001	18.4%	< .0001	16.5%	< .0001	17.8%	< .0001	22.2%	< .0001	29.2%
**Prior stroke/SE**	11.3%	< .0001	11.1%	< .0001	10.1%	< .0001	9.4%	< .0001	13.2%	< .0001	14.3%
**Obesity**	21.2%	< .0001	21.4%	< .0001	19.9%	< .0001	21.1%	< .0001	21.5%	< .0001	15.3%
**Congestive heart failure**	26.5%	< .0001	25.7%	< .0001	22.1%	< .0001	22.8%	< .0001	31.2%	< .0001	34.7%
**Diabetes**	38.1%	< .0001	37.0%	< .0001	37.2%	< .0001	36.3%	< .0001	41.1%	< .0001	39.2%
**Hypertension**	89.1%	< .0001	89.5%	< .0001	88.5%	0.0018	88.6%	< .0001	89.3%	< .0001	87.9%
**Chronic obstructive pulmonary disease**	23.4%	< .0001	22.7%	< .0001	21.5%	< .0001	22.3%	< .0001	25.4%	< .0001	31.3%
**Renal disease**	23.7%	< .0001	24.5%	< .0001	16.8%	< .0001	18.1%	< .0001	28.4%	< .0001	33.6%
**Myocardial Infarction**	13.2%	< .0001	12.9%	< .0001	10.7%	< .0001	11.1%	< .0001	15.7%	< .0001	17.5%
**Dyspepsia or stomach discomfort**	19.7%	< .0001	19.6%	< .0001	18.1%	< .0001	19.1%	< .0001	20.5%	< .0001	25.0%
**Peripheral vascular disease**	45.4%	< .0001	43.9%	< .0001	42.4%	< .0001	42.9%	< .0001	49.7%	< .0001	53.6%
**Transient ischemic attack**	8.4%	< .0001	9.6%	0.0373	7.6%	< .0001	7.3%	< .0001	8.0%	< .0001	9.4%
**Coronary artery disease**	41.7%	< .0001	41.9%	< .0001	38.6%	< .0001	39.1%	< .0001	44.3%	< .0001	48.0%
**History of falls**	6.9%	< .0001	7.2%	< .0001	5.4%	< .0001	6.4%	< .0001	7.4%	< .0001	13.0%
**Baseline medication usage**											
ACE/ARB	59.4%	< .0001	59.9%	< .0001	60.9%	< .0001	59.4%	< .0001	58.6%	< .0001	51.2%
Amiodarone	1.5%	< .0001	1.5%	< .0001	1.7%	< .0001	1.6%	< .0001	1.5%	< .0001	2.6%
Beta blockers	48.4%	< .0001	48.2%	< .0001	48.8%	< .0001	47.5%	< .0001	49.1%	< .0001	43.2%
H2-receptor antagonist	6.4%	< .0001	6.5%	< .0001	5.9%	< .0001	6.1%	< .0001	6.8%	< .0001	8.4%
Proton pump inhibitor	29.1%	< .0001	29.5%	< .0001	27.8%	< .0001	28.7%	< .0001	29.4%	< .0001	32.5%
Statins	55.8%	< .0001	56.4%	< .0001	55.9%	< .0001	55.4%	< .0001	55.4%	< .0001	49.1%
Anti-platelets	14.1%	< .0001	14.4%	< .0001	13.1%	< .0001	13.1%	< .0001	14.6%	< .0001	16.4%
NSAIDS	23.5%	< .0001	23.6%	< .0001	25.0%	< .0001	25.1%	< .0001	21.7%	< .0001	20.3%
**Baseline all-cause healthcare utilizations**											
Inpatient Admission Visit	48.8%	< .0001	48.1%	< .0001	41.7%	< .0001	44.5%	< .0001	54.2%	< .0001	62.2%

*Cohorts are assigned based on first observed OAC treatment post-diagnosis.

^†^Edoxaban (N = 798) was not included because of sample size concerns.

^‡^as the INR value is not available in the databases, a modified HAS-BLED score was calculated with a range of 0 to 8.

ACE/ARB- Angiotensin Converting Enzyme Inhibitors/Angiotensin Receptor Blockers; CHA_2_DS_2_-VASc- Congestive heart failure, Hypertension, Age ≥ 75 years, Diabetes mellitus, Stroke, Vascular disease, Age 65–74 years, Sex category; HAS-BLED- Hypertension, Abnormal Renal or Liver Function, Stroke, Bleeding History or Predisposition, Labile International Normalized Ratios, Elderly, Drugs or Alcohol; H2- Histamine Type-2; NSAIDs- Non-steroidal Anti-inflammatory Drugs; OAC- Oral Anticoagulants; SD- Standard Deviation; SE- Systemic Embolism.

### Clinical outcomes

**Fig 2:**
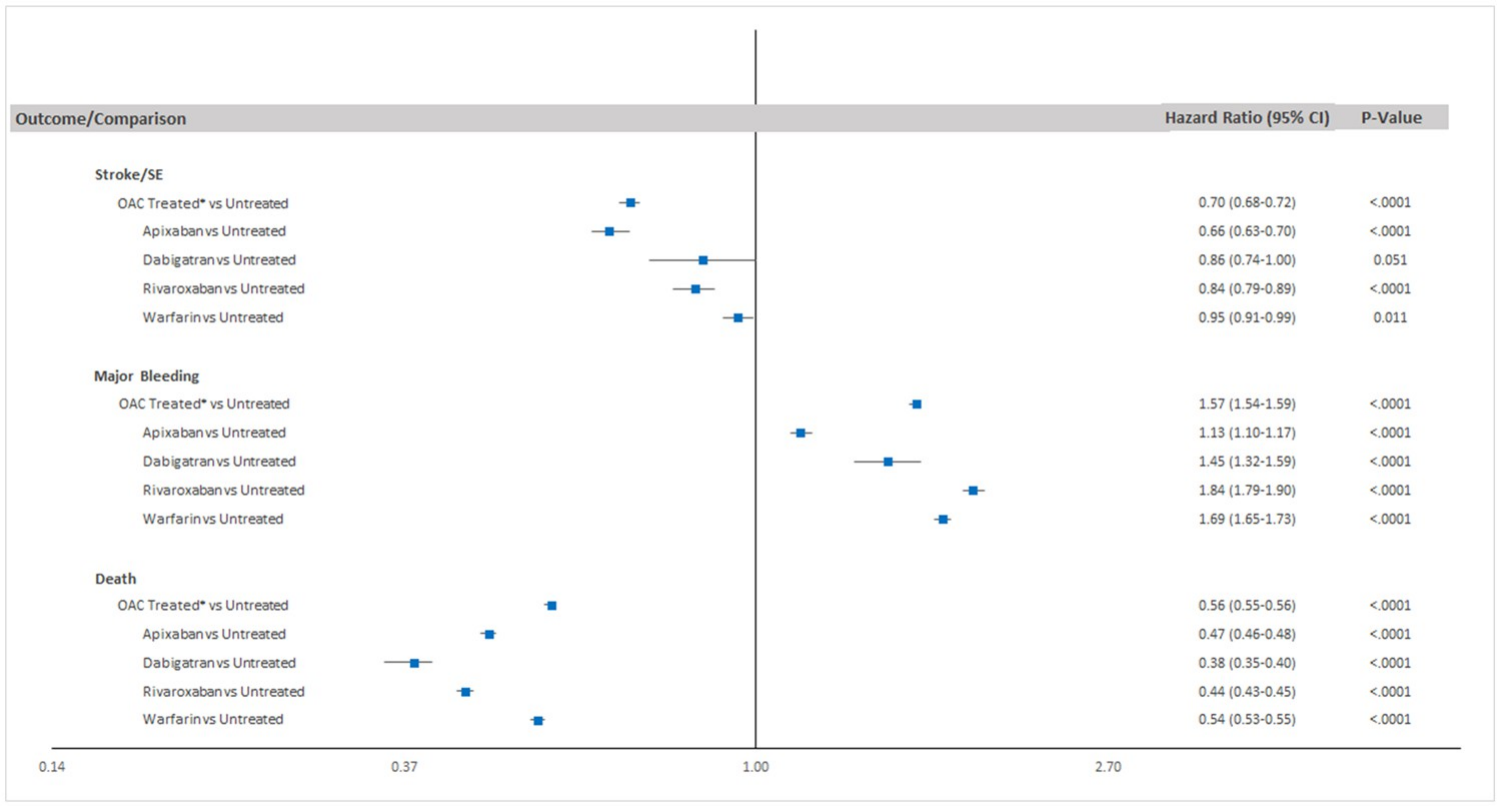
Adjusted risk of stroke/SE, major bleeding, and all-cause death based on OAC treatment after first atrial fibrillation diagnosis. Republished from Munir MB, Hlavacek P, Keshishian A, et al. Contemporary clinical and economic outcomes among oral anticoagulant treated and untreated elderly patients with atrial fibrillation. Heart Rhythm. 2021;18(8, S1 File): S431. doi: https://doi.org/10.1016/j.hrthm.2021.06.1064 under a CC BY license, with permission from Elsevier, original copyright 2021. *OAC Treated cohort includes apixaban, dabigatran, edoxaban, rivaroxaban, and warfarin treated patients; CI- Confidence Interval; OAC- Oral Anticoagulant; SE- Systemic Embolism.

In the regression analysis modeling OAC treatment, patients who were OAC treated had a lower risk of stroke/SE (Hazard Ratio [HR]: 0.70; 95% Confidence Interval [CI]: 0.68–0.72) compared to patients without OAC treatment (Fig 2). Apixaban (HR: 0.66, 95% CI: 0.63–0.70), rivaroxaban (HR: 0.84, 95% CI: 0.79–0.89), and warfarin (HR: 0.95, 95% CI: 0.91–0.99) were associated with a lower risk of stroke/SE compared to patients without OAC treatment, while dabigatran (HR: 0.86, 95% CI: 0.74–1.00) was associated with a similar risk of stroke/SE. Similar trends were observed for ischemic stroke (S1 Fig in S1 File). OAC treated patients were at a higher risk of having a major bleed (HR: 1.57; 95% CI: 1.54–1.59) during their treatment period compared with the OAC untreated patients. Consistent trends were observed across all individual OACs (apixaban HR: 1.13; 95% CI: 1.10–1.17; dabigatran HR: 1.45; 95% CI: 1.32–1.59; rivaroxaban HR: 1.84; 95% CI: 1.79–1.90; warfarin HR: 1.69; 95% CI: 1.65–1.73) for the major bleeding outcome. Generally consistent trends were observed for GI bleeding and intracranial hemorrhage across various OAC treated patients compared to patients without OAC treatment; however, there was no significant difference between apixaban and dabigatran treated and OAC untreated patients for intracranial hemorrhage (S1 Fig in S1 File). OAC treated AF patients in our cohort experienced a lower risk of all-cause death compared to OAC untreated patients (HR: 0.56; 95% CI: 0.55–0.56) with consistent trends across all the OACs.

### Economic outcomes

OAC treated patients had significantly lower all-cause healthcare costs PPPM compared to OAC untreated patients ($4,381 vs $7,172; p<0.0001). This trend was apparent for all individual OACs (apixaban: $4,110 vs $6,719; dabigatran: $3,919 vs $6,710; rivaroxaban: $4,111 vs $6,728; warfarin: $4,608 vs $7,127; all p<0.001). While inpatient and other costs were significantly lower for OAC treated patients versus untreated patients, outpatient and pharmacy costs were significantly higher for the OAC treated patients compared to OAC untreated patients.

Stroke/SE-related hospitalization costs PPPM were significantly lower for all OAC treated patients ($31 vs $41, <0.0001) and those treated with apixaban ($21 vs $43; p<0.0001), dabigatran ($30 vs $45; p- 0.0104), and rivaroxaban ($27 vs $43, p<0.0001). Major bleeding-related hospitalization costs PPPM were significantly higher in the OAC treated cohort ($80 vs $60, p<0.0001) and for those treated with rivaroxaban ($81 vs $66, p<0.0001) and warfarin ($104 vs $67, p<0.0001); however, the costs were significantly lower for those treated with apixaban ($53 vs $65; p<0.0001; Table 2).

**Table 2:** Comparison of per patient per month adjusted healthcare costs between OAC treated and untreated patients after first atrial fibrillation diagnosis*.

	OAC Treated vs Untreated		Apixaban vs Untreated		Dabigatran vs Untreated		Rivaroxaban vs Untreated		Warfarin vs Untreated	
Variable	Marginal Effect	p-value	Marginal Effect	p-value	Marginal Effect	p-value	Marginal Effect	p-value	Marginal Effect	p-value
**Total healthcare costs**	$4,381 vs $7,172	< .0001	$4,110 vs $6,719	< .0001	$3,919 vs $6,710	< .0001	$4,111 vs $6,728	< .0001	$4,608 vs $7,127	< .0001
**Medical costs**	$3,761 vs $6,881	< .0001	$3,367 vs $6,423	< .0001	$3,228 vs $6,411	< .0001	$3,418 vs $6,434	< .0001	$4,201 vs $6,834	< .0001
**Inpatient**	$1,919 vs $4,170	< .0001	$1,675 vs $3,743	< .0001	$1,617 vs $3,699	< .0001	$1,732 vs $3,751	< .0001	$2,153 vs $4,061	< .0001
**Outpatient**	$1,081 vs $935	< .0001	$984 vs $886	< .0001	$951 vs $856	< .0001	$980 vs $877	< .0001	$1,180 vs $877	< .0001
**Other costs**	$772 vs $1,764	< .0001	$675 vs $1,792	< .0001	$596 vs $1,855	< .0001	$674 vs $1,804	< .0001	$937 vs $1,883	< .0001
**Pharmacy Costs**	$664 vs $288	< .0001	$775 vs $295	< .0001	$749 vs $299	< .0001	$754 vs $293	< .0001	$469 vs $289	< .0001
**Major bleeding hospitalization costs**	$80 vs $60	< .0001	$53 vs $65	< .0001	$63 vs $70	0.1621	$81 vs $66	< .0001	$104 vs $67	< .0001
**Stroke/SE hospitalization costs**	$31 vs $41	< .0001	$21 vs $43	< .0001	$30 vs $45	0.0104	$27 vs $43	< .0001	$44 vs $45	0.7576

*Patients were followed until the earliest of death, disenrollment, discontinuation, switch, or end of study.

OAC- Oral Anticoagulant; SE- Systemic Embolism.

### Sensitivity analyses

We also conducted three sensitivity analyses. In the first two sensitivity analyses, where death was treated as a competing risk and patients were censored at one-year follow-up, the results showed consistent trends. (S2 and S3 Figs in S1 File). In the third sensitivity analysis, we tested 3 falsification outcomes to determine if there was a difference between OAC treatment versus no OAC treatment for clinically unrelated outcomes of COPD, pneumonia and UTI hospitalizations. Falsification analysis for UTI was statistically significant for the comparisons examined using cox regression models, which implies that there may be residual confounding (S4 Fig in S1 File).

## Discussion

In this large and contemporary cohort of elderly AF patients stratified based on OAC treatment status after their initial AF diagnosis, we report several key findings [1]. The prevalence of OAC treatment is still low at 41% in elderly AF patient as 59% of patients were not treated with an OAC after the first AF diagnosis [2]. The adjusted risk of stroke/SE and all-cause death was lower in AF patients on OAC treatment compared to patients who were not treated with an OAC. This trend was by and large uniform across all the OACs [3]. The adjusted risk of major bleeding was higher in AF patients on OAC treatment compared to AF patients who were not treated with an OAC. However, among the OAC treated group, the adjusted risk of GI bleeding was lower in patients treated with apixaban while the adjusted risk of intracranial hemorrhage was lower in patients treated with apixaban and dabigatran [4]. The total adjusted healthcare cost PPPM was lower in the OAC treated group when compared to AF patients not treated with an OAC. Similar trends were observed for all the OACs analyzed in our study.

One of the most dreaded complications of AF is stroke and AF associated strokes are more debilitating when compared to strokes not related to AF [2, 3]. OACs are now the standard of care for mitigation of stroke risk in eligible AF patients [26]. Our results showed that the adjusted risk of hard clinical outcomes such as stroke/SE and mortality was lower in AF patients treated with an OAC versus AF patients not treated with an OAC. Few earlier studies have assessed the hard-clinical endpoints in AF patients based on OAC treatment status in a real-world contemporary cohort predominated by DOAC treatment. In a study of more than 39,000 AF patients insured by Medicare, Hernandez et al. [10] showed that adherent use of an OAC was associated with a lower risk of stroke (HR 0.62, 95% CI 0.52–0.74). They also found no significant difference in the risk of stroke in AF patients who are adherent to either a DOAC or warfarin (HR 0.77, 95% CI 0.56–1.04). Similarly, in a study of more than 64,000 AF patients from a large US commercial insurance database with 87,157 patient-years of follow-up, Yao et al. [11] showed that approximately 1,150 patients had an ischemic stroke or SE. They further demonstrated that AF patients with a CHA_2_DS_2_-VASc score of 2 or 3 have a significant increased risk of stroke if they were not treated with oral anti-coagulation for more than 6 months compared to patients who were treated with oral anti-coagulation (HR 2.73, 95% CI 1.76–4.23). In their study, the association between stroke and non-adherent use of an OAC became stronger in AF patients at 6 months of follow-up with further elevated CHA_2_DS_2_-VASc score of 4 (HR 3.66, 95% CI 2.68–5.01). In another study of 2,882 patients from the Veterans Health Administration database, Borne et al. [12] demonstrated that in AF patients not on dabigatran, the risk of stroke and mortality was significantly higher when compared to AF patients who are adherent to dabigatran (HR 1.07, 95% CI 1.03–1.12). Our real-world contemporary cohort of elderly AF patients largely corroborated the findings of these earlier studies as it also depicted lower risk of stroke and mortality in patients who were subjected to an OAC treatment versus patients who were not treated with an OAC after the first AF diagnosis.

Our results also demonstrated that adjusted risk of major bleeding was higher in AF patients on OAC treatment versus AF patients who were never prescribed an OAC. Additionally, while all OACs were associated with a higher risk of GI bleeding, apixaban patients had the smallest magnitude of increase when compared to OAC untreated patients. Several earlier studies have shown safety of apixaban with respect to lower GI bleeding event rates when compared to other OACs. In a nationwide study of AF patients on OACs from Taiwan, Chan et al. [27] demonstrated that standard dose apixaban was associated with a lower prevalence of GI bleeding when compared to warfarin (HR 0.32, 95% CI 0.21–0.49). Similarly, in another study of approximately 1.6 million AF patients from the Medicare beneficiaries database, Ray et al. [28] demonstrated that the adjusted incidence of hospitalization for significant upper GI tract bleeding was lower in patients who were prescribed apixaban when compared to other OACs (incidence rate ratio [IRR] 0.61, 95% CI 0.52–0.70 when compared to dabigatran and IRR of 0.64, 95% CI 0.57–0.73 when compared to warfarin). In another study of more than 370,000 AF patients from the large administrative claims-based database, Abraham et al. [29] showed that the adjusted risk of GI bleeding was lower in patients who were treated with apixaban when compared to dabigatran (HR 0.39, 95% CI 0.27–0.58) and rivaroxaban (HR 0.33, 95% CI 0.25–0.61). This difference of significantly lower GI bleeding with apixaban persisted across all age-groups in their cohort.

Our results also demonstrated that the total healthcare adjusted costs PPPM were significantly lower in AF patients who were OAC treated versus those that were untreated. This effect was consistent across all the OACs and in different clinical settings with the predominant exception of the pharmacy costs which were higher in OAC treated AF patients. Additionally, in Table 2 each individual DOAC treated (apixaban, dabigatran, rivaroxaban) cohort had lower medical costs than the warfarin treated cohort, suggesting that DOACs have lower costs than warfarin overall. This aligns with the findings of recent studies that indicated lower costs for DOACs versus warfarin [30–33]. Earlier studies have reported increased healthcare costs in patients after the AF diagnosis primarily after they suffered a stroke; however, no large studies have stratified such outcomes in AF patients based on OAC treatment status. In a cohort of approximately 3,891 AF patients insured by private payers, Boccuzzi et al. [13] demonstrated that the average PPPM costs were significantly higher at $1,235 after the AF diagnosis when compared to the average PPPM costs of $412 before the onset of AF. They also showed that the healthcare costs PPPM were higher in AF patients who suffered a stroke and ranged from $2,235 to $3,135. Similarly, in a study of 568 patients with a history of incident and recurrent strokes, Hannon et al. [14] demonstrated that the individual healthcare costs at 2-years of follow-up were substantially higher in patients in whom the stroke was associated with AF as compared to patients with stroke not related to AF (median costs $36,865 vs. $18,691, respectively). They also showed that the individual patient costs for inpatient and nursing home stays were significantly higher when strokes were associated with AF. In a study of 9,455 Medicare patients from 2006–2008, Fitch et al. [15] demonstrated that the healthcare costs per patient were $63,781 after the diagnosis of an ischemic stroke compared to the healthcare costs of $35,474 in patients without an ischemic stroke in AF patients. In the current study, we have reported for the first time that the adjusted total healthcare costs were lower in AF patients who were treated with an OAC compared to AF patients who were never prescribed an OAC. Similarly, stroke/SE associated healthcare costs were also lower in OAC treated AF patients when compared to OAC untreated patients. These findings have important implications in the management of AF patients as they demonstrate that utilization of OAC treatment in such patients is cost-effective overall and should provide an incentive against frequent insurance denials especially for the DOACs [34].

The study has several important limitations that should be considered while interpreting the results. As this is a retrospective study, only associations and not causations can be inferred. The study data were collected for administrative purposes and not research, meaning that the presence of a claim for a filled prescription does not necessarily indicate that the medication was taken as prescribed. In addition, coding errors and lack of granular clinical data could have introduced bias into the study. For example, while we adjusted for renal disease and included HAS-BLED scores as time-varying covariates, the clinical information on kidney disease staging is not captured in Medicare dataset and hence could not be utilized in our study [16]. Over-the-counter aspirin use and laboratory data, including the international normalized ratio (INR) were not available in the database and hence could not be used in the study. We only evaluated the first episode of treatment, so if a patient discontinued or switched OACs, we did not evaluate subsequent outcomes. In addition, although weights were used to balance cohorts, potential residual confounding may exist. From our falsification sensitivity analysis, we did observe significant associations for one of the outcomes, which implies that there may be residual confounding [25]. Lastly, the Medicare database only includes patients who have fee-for-service Medicare insurance; therefore, the results may not be generalizable to patients without insurance or those with other insurance (e.g. Medicare Advantage, commercial, VA).

## Conclusion

In our large, contemporary sample of AF patients from the Medicare database, we demonstrated that the adjusted risk of stroke/SE and all-cause death was lower in OAC treated versus OAC untreated patients, while the adjusted risk of major bleeding was higher in OAC treated AF patients. The adjusted total healthcare costs were lower in OAC treated AF patients.

## Supporting information

S1 File Supplemental material. (DOCX)
