# Supplementary material for: Contemporary clinical and economic outcomes among oral anticoagulant treated and untreated elderly patients with atrial fibrillation: Insights from the United States Medicare database
Source: PLoS One. 2022 Feb 17;17(2):e0263903. doi: 10.1371/journal.pone.0263903 (PMC8853505; doi:10.1371/journal.pone.0263903)
Supplement: S1 File — (DOCX) [file pone.0263903.s001.docx]

**S1 File. Supplemental Material**

**S1 Fig. Adjusted Risk of Ischemic Stroke, Gastrointestinal Bleeding, and Intracranial Hemorrhage Based on OAC Treatment Status After First Atrial Fibrillation Diagnosis**


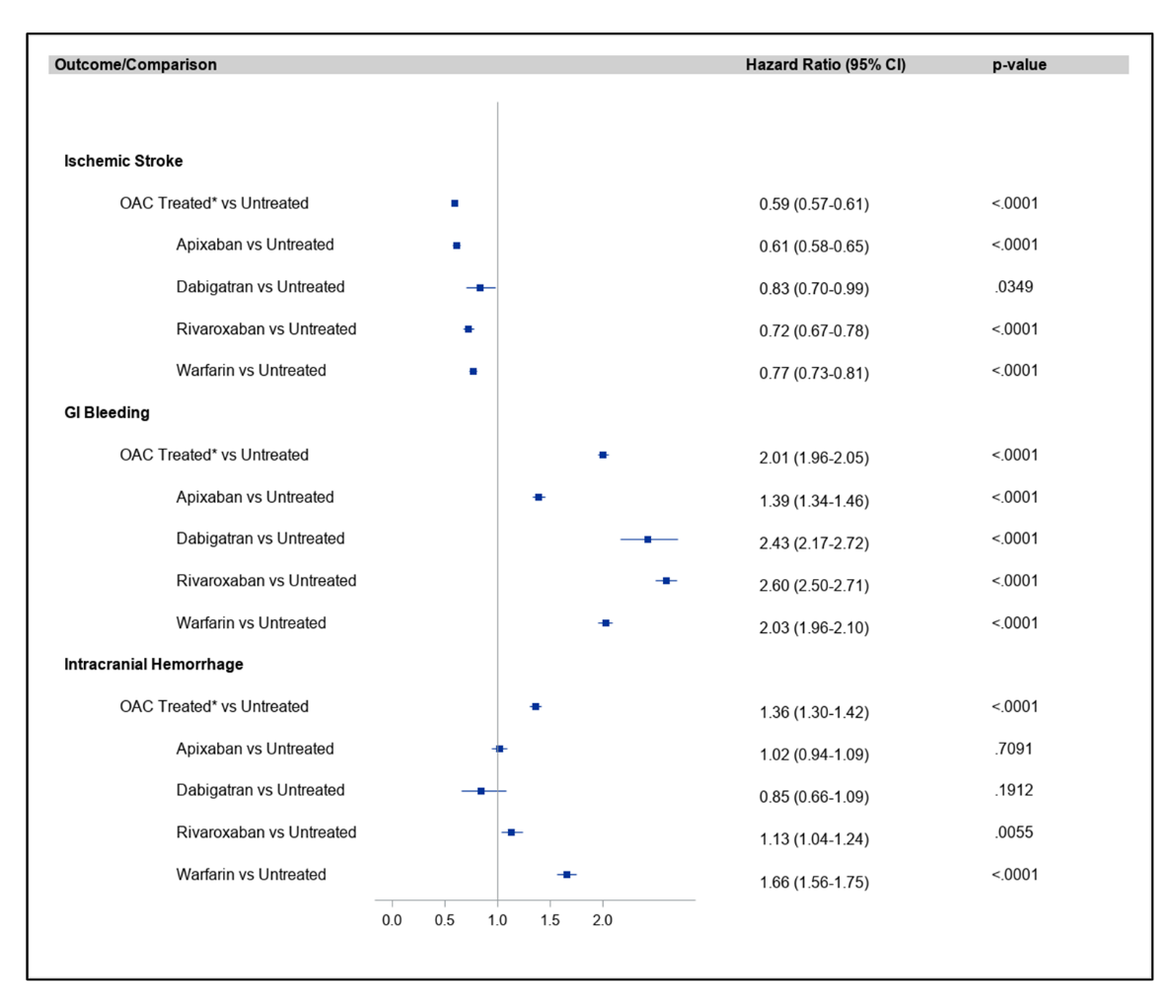


*OAC Treated cohort includes apixaban, dabigatran, edoxaban, rivaroxaban, and warfarin treated patients.

CI: Confidence Interval; GI: Gastrointestinal; OAC: Oral Anticoagulant; SE: Systemic Embolism

**S2 Fig. Sensitivity Analysis: Adjusted Risk of Stroke/SE, Major Bleeding, and Death Based on OAC Treatment Status After First Atrial Fibrillation Diagnosis with Death as a Competing Risk**
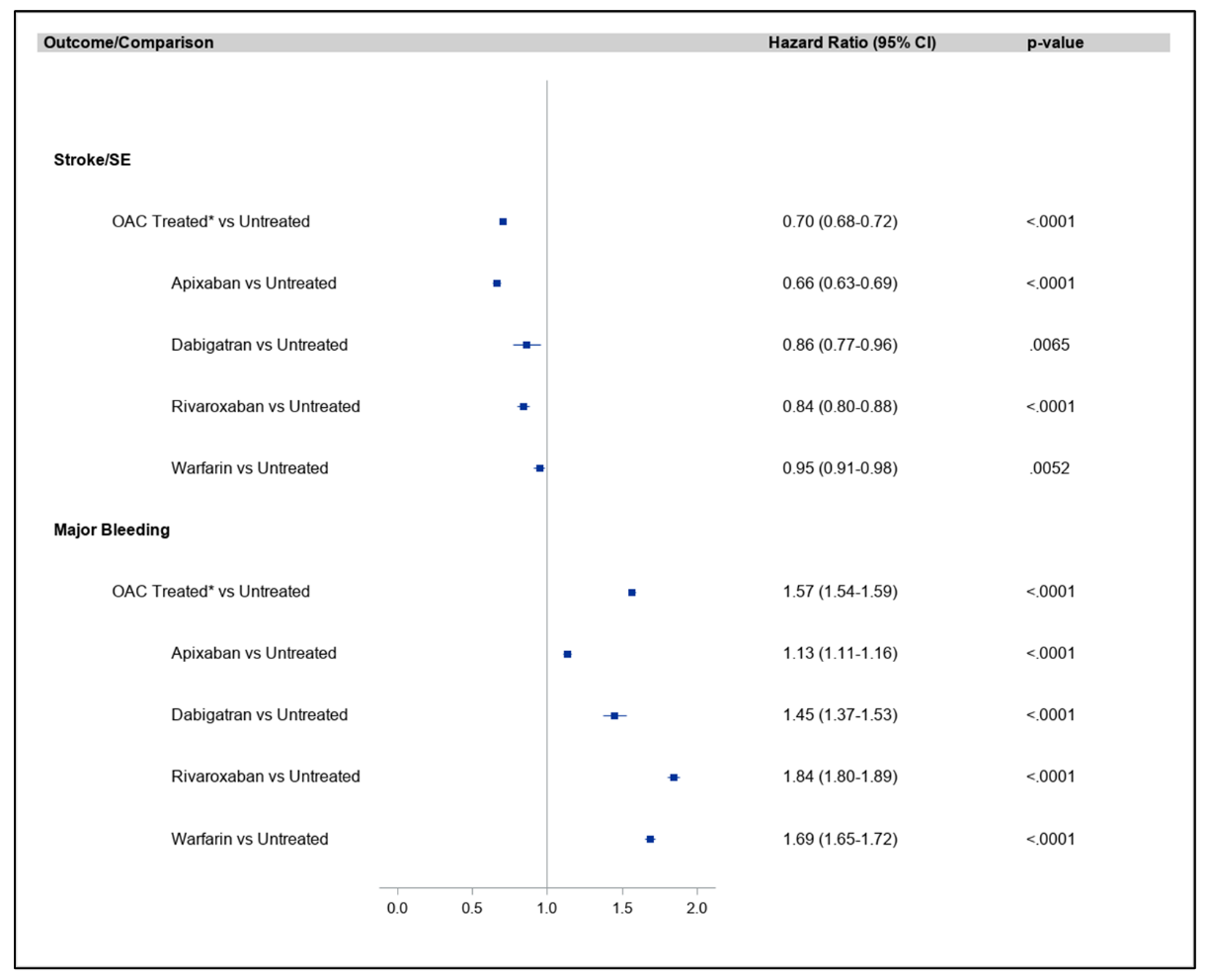


*OAC Treated cohort includes apixaban, dabigatran, edoxaban, rivaroxaban, and warfarin treated patients.

CI: Confidence Interval; OAC: Oral Anticoagulant; SE: Systemic Embolism

**S3 Fig. Adjusted Risk of Stroke/SE, Major Bleeding, and Death Based on OAC Treatment After First Atrial Fibrillation Diagnosis - Censored at One Year**


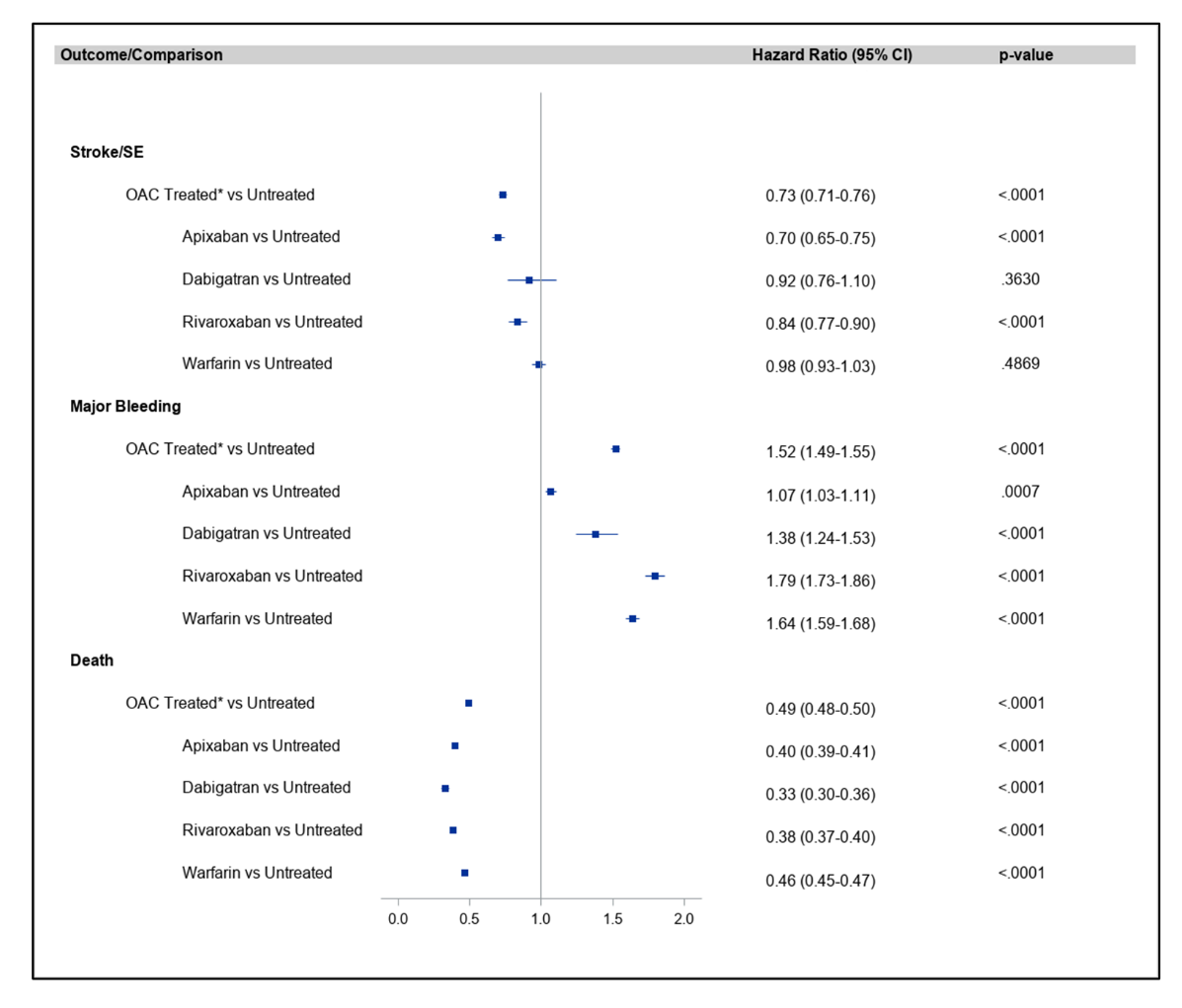


*OAC Treated cohort includes apixaban, dabigatran, edoxaban, rivaroxaban, and warfarin treated patients.

CI: Confidence Interval; OAC: Oral Anticoagulant; SE: Systemic Embolism

**S4 Fig. Adjusted Risk of Falsification Outcomes Based on OAC Treatment Status**


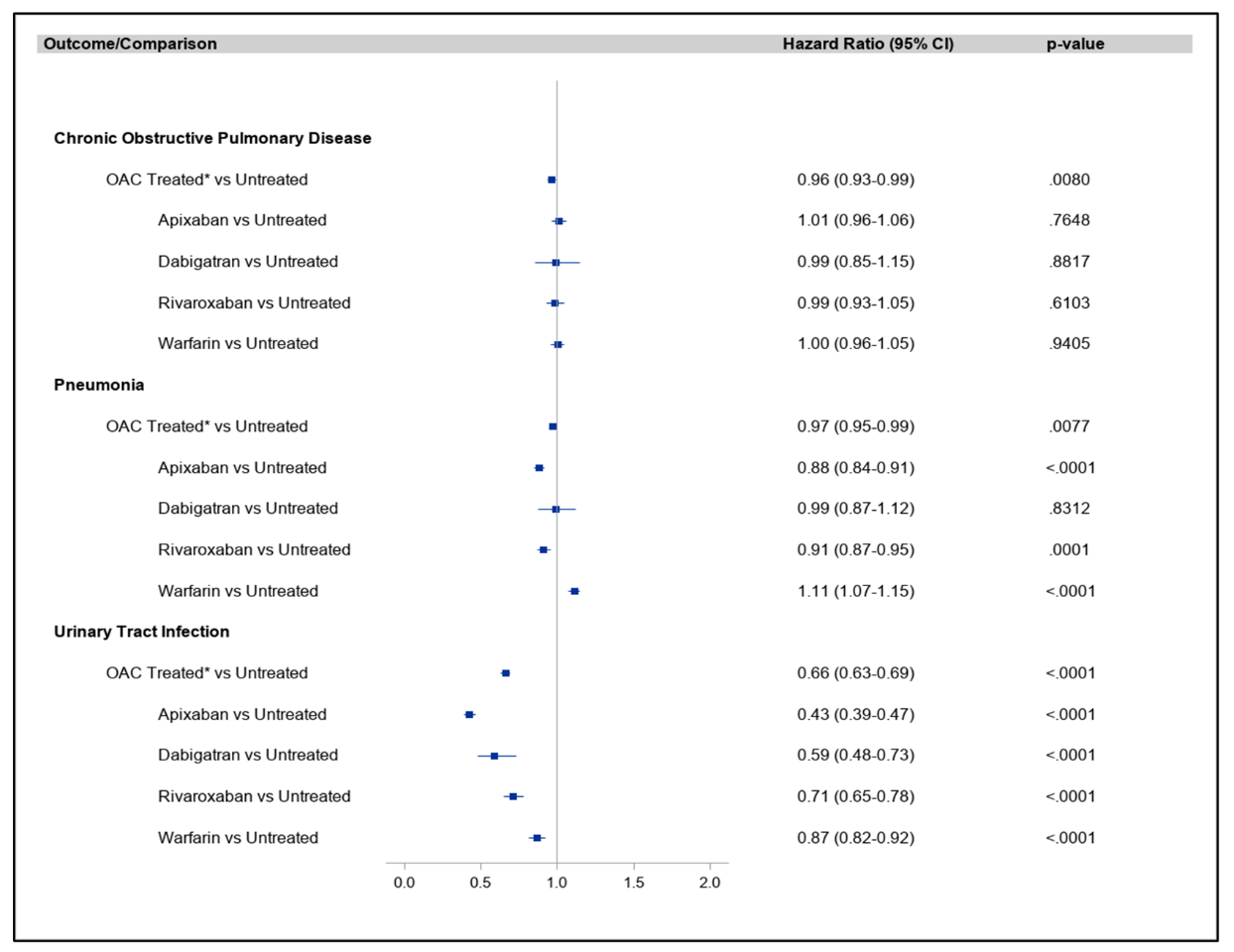


*OAC Treated cohort includes apixaban, dabigatran, edoxaban, rivaroxaban, and warfarin treated patients.

CI: Confidence Interval; OAC: Oral Anticoagulant; SE: Systemic Embolism
